# Supplementary material for: Air Pollution-Related Respiratory Diseases and Associated Environmental Factors in Chiang Mai, Thailand, in 2011–2020
Source: Trop Med Infect Dis. 2022 Oct 31;7(11):341. doi: 10.3390/tropicalmed7110341 (PMC9696662; doi:10.3390/tropicalmed7110341)
Supplement: Supplementary file 1 [file tropicalmed-07-00341-s001.zip › tropicalmed-1928766-supplementary.pdf]

Tropical Medicine and Infectious Disease

MDPI

Supplementary Materials:

Air Pollution-Related Respiratory Diseases and Associated Environmental Factors in Chiang Mai, Thailand, in 2011–2020

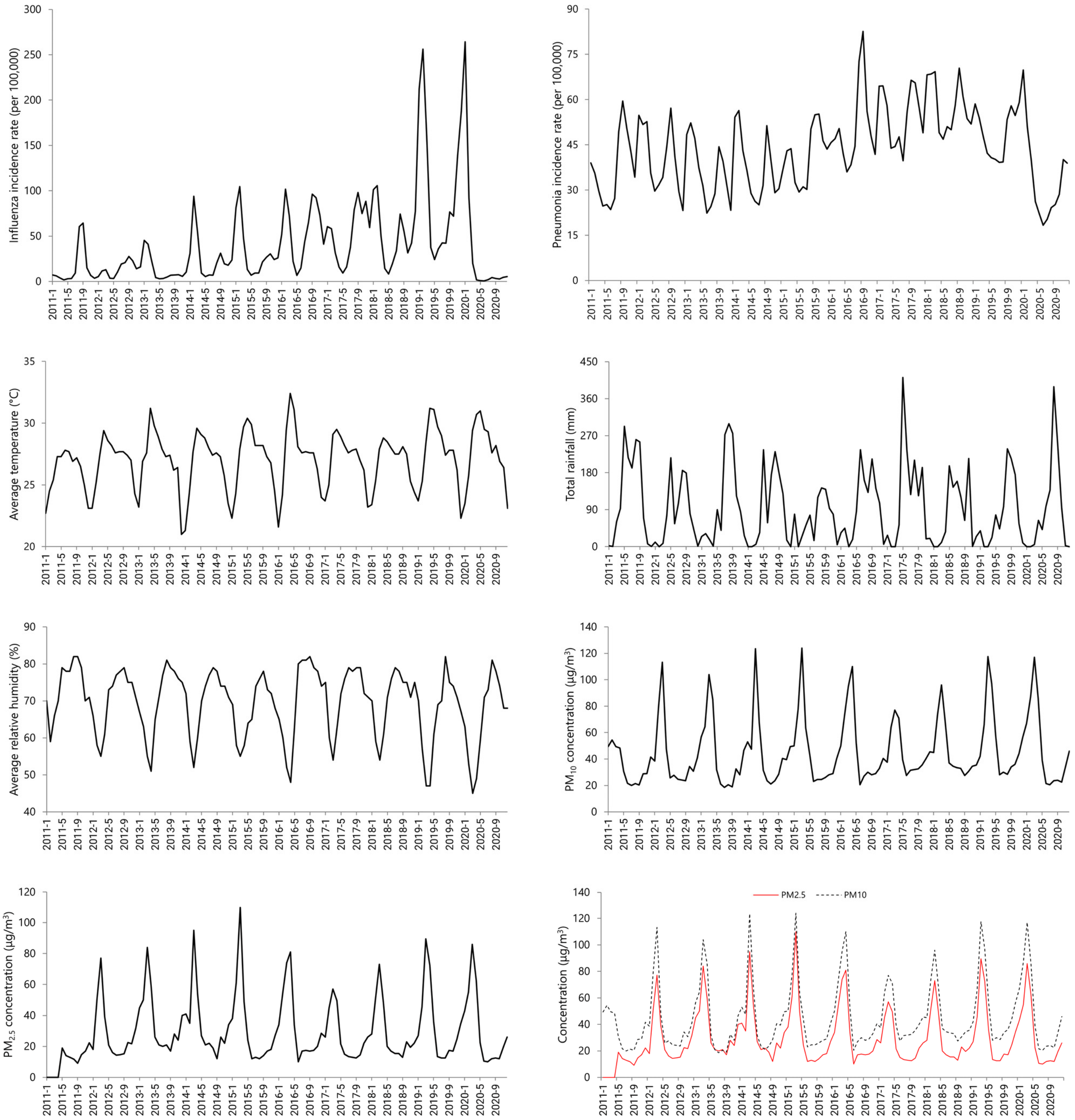

Figure S1. Monthly incidence of influenza and pneumonia, climate data, and concentration of air pollutants in Chiang Mai province from 2011 to 2020. (From January to April 2011, $PM_{2.5}$ concentration data was unavailable.)

Table S1. Baseline information of monthly meteorological and air pollutant data in Chiang Mai from 2011 to 2020.

Season	Dry, Cool		Hot			Rainy					Dry, Cool	
**Month**	**Jan**	**Feb**	**Mar**	**Apr**	**May**	**Jun**	**Jul**	**Aug**	**Sep**	**Oct**	**Nov**	**Dec**
Temperature (°C)												
Station 1 [a]	22.9	25.1	28.0	30.0	29.6	28.7	28.0	27.6	27.8	27.2	26.1	23.3
Station 2 [b]	15.9	18.9	21.5	22.7	22.1	20.7	20.0	19.8	19.9	18.7	18.0	15.5
Mean difference ± SE	**6.9 ± 0.4**	**6.2 ± 0.4**	**6.6 ± 0.6**	**7.3 ± 0.7**	**7.5 ± 0.6**	**8.0 ± 0.3**	**8.0 ± 0.2**	**7.8 ± 0.1**	**7.9 ± 0.1**	**8.5 ± 0.2**	**8.2 ± 0.3**	**7.8 ± 0.5**
Relative humidity (%)												
Station 1	68.7	58.6	53.5	56.8	67.8	73.4	76.5	79.6	78.5	76.0	72.7	71.1
Station 2	70.5	53.5	48.8	59.4	76.5	88.4	91.3	93.0	91.6	90.6	85.2	81.3
Mean difference ± SE	-1.8 ± 3.1	5.1 ± 2.6	4.7 ± 3.1	-2.6 ± 3.9	**-8.7 ± 2.9**	**-15.0 ± 1.7**	**-14.8 ± 1.2**	**-13.4 ± 1.0**	**-13.1 ± 0.9**	**-14.6 ± 1.2**	**-12.5 ± 1.4**	**-10.2 ± 2.0**
Total precipitation (mm) [c]												
Station 1	21.8	7.8	13.5	45.2	172.4	114.4	154.9	220.9	189.4	131.5	40.9	9.4
Station 2	24.7	21.7	47.4	87.1	242.0	178.3	318.9	375.4	297.1	195.0	50.5	24.0
Mean difference ± SE	-2.8 ± 8.5	-13.9 ± 12.5	-33.9 ± 16.9	**-41.9 ± 18.0**	-69.6 ± 50.7	-63.9 ± 36.1	**-164 ± 40.0**	**-154 ± 41.3**	**-108 ± 38.6**	-63.5 ± 43.0	-9.6 ± 15.0	-14.6 ± 8.8
$PM_{2.5}$ (μg/m^3) [d]												
Station 1	33.3	51.5	81.0	62.3	26.5	15.0	15.6	16.4	17.0	19.0	23.8	30.6
Station 2	32.3	48.7	80.8	55.5	24.5	14.6	14.1	13.8	12.7	17.6	19.5	27.6
Mean difference ± SE	0.9 ± 5.6	2.8 ± 4.2	0.2 ± 9.6	6.8 ± 7.7	2.0 ± 3.8	0.4 ± 2.4	1.5 ± 2.2	2.7 ± 1.9	**4.3 ± 1.5**	1.4 ± 3.1	4.3 ± 1.4	3.0 ± 3.4
PM_{10} (μg/m^3)												
Station 1	48.9	69.6	104.6	77.6	40.2	23.3	23.8	25.0	25.4	30.7	33.0	44.0
Station 2	48.9	67.6	98.8	71.1	37.5	26.4	26.4	27.2	27.3	32.9	35.3	44.6
Mean difference ± SE	0.0 ± 4.4	2.0 ± 6.0	5.8 ± 11.2	6.4 ± 9.7	2.7 ± 5.0	-3.1 ± 2.5	-2.6 ± 2.6	-2.3 ± 2.4	-1.9 ± 2.7	-2.2 ± 2.8	-2.3 ± 3.0	-0.6 ± 3.2

[a] Station 1, weather station#327501 (meteorological data) or air quality monitoring station#35T ($PM_{2.5}$ and PM_{10} data). [b] Station 2, weather station#327202 or air quality monitoring station#36T. [c] Total precipitation in Thailand only refers to total rainfall. [d] From January to April of 2011, $PM_{2.5}$ data was unavailable, and from 2011 to June of 2016, data was only retrieved from a 36T station. Values in bold were indicated significantly difference between two stations (p-value < 0.05). Due to significant different of the meteorological data between two stations, only the data from station#327501 were used in this study.

Table S2. Details of district hospitals/health service centers in health service network of air pollution-related illness.

District	Hospital/Health Service Center	Decimal Degree		UTM Coordinates	
		**Latitude**	**Longitude**	**X**	**Y**
Muang	Central Chiangmai Memorial Hospital	18.773001	98.998464	499838.118	2075710.821
	Changpuek Hospital	18.799122	98.986928	498622.531	2078601.035
	Chiangmai Klaimor Hospital	18.760717	98.972118	497061.253	2074351.887
	Chiangmai Medical Center Hospital	18.780418	98.988318	498768.867	2076531.515
	Fort Kawila Hospital	18.777927	99.014745	501553.957	2076255.922
	Maharaj Nakorn Chiang Mai Hospital	18.789854	98.974354	497297.391	2077575.718
	Municipal Hospital	18.788495	99.001807	500190.426	2077425.158
	Nong Pa Khrang Municipal Health Service Center	18.786313	99.033687	503550.055	2077184.065
	Siamrad Chiangmai Hospital	18.810737	98.981411	498041.308	2079886.234
Chom Thong	Chomthong Hospital	18.406576	98.674273	465597.423	2035199.359
Mae Chaem	Mae Chaem Hospital	18.498260	98.365150	432983.290	2045430.322
Chiang Dao	Chiang Dao Hospital	19.404025	98.974549	497327.816	2145533.105
Doi Saket	Doi Saket Hospital	18.868295	99.128654	513551.446	2086259.608
Mae Taeng	Mae Taeng Hospital	19.137169	98.960362	495831.535	2116005.484
Mae Rim	Nakornping Hospital	18.851413	98.966402	496460.687	2084387.095
Samoeng	Samoeng Hospital	18.846905	98.732530	471823.085	2083909.213
Fang	Fang Hospital	19.913784	99.206226	521584.074	2201953.921
Mae Ai	Mae Ai Hospital	20.038933	99.302212	531605.288	2215818.205
Phrao	Phrao Hospital	19.367586	99.200052	521008.819	2141513.006

San Pa Tong	San Pa Tong Hospital	18.595356	98.885024	487869.832	2056059.339
San Kamphaeng	San Kamphaeng Hospital	18.716174	99.122054	512867.801	2069427.608
San Sai	Sansai Hospital	18.920922	98.994147	499383.682	2092077.693
Hang Dong	Hang Dong Hospital	18.683074	98.918362	491391.471	2065762.851
Hod	Hod Hospital	18.179120	98.611242	458886.555	2010046.383
Doi Tao	Doi Tao Hospital	17.950273	98.690729	467250.467	1984711.195
Omkoi	Omkoi Hospital	17.802557	98.361002	432277.965	1968456.899
Saraphi	Sarapee Hospital	18.680880	99.043184	504553.706	2065518.683
Wiang Haeng	Wiang Haeng Hospital	19.560225	98.635756	461793.278	2162857.662
Chai Prakan	Chai Prakan Hospital	19.705471	99.136848	514341.463	2178895.040
Mae Wang	Mae Wang Hospital	18.615488	98.773641	476121.502	2058297.983
Mae On	Mae On Hospital	18.744628	99.221096	523305.645	2072585.942
Doi Lo	Doi Lo Hospital	18.477639	98.780191	476793.849	2043045.085
Kalayaniwattana	Watchan Community Hospital	19.077636	98.315840	428024.341	2109558.200

Table S3. Incidence rates of influenza and pneumonia in Chiang Mai from 2011 to 2020.

Month	Incidence Rate of Influenza (per 100,000 Population)									
	**2011**	**2012**	**2013**	**2014**	**2015**	**2016**	**2017**	**2018**	**2019**	**2020**
Jan	7.2	5.3	45.5	30.9	81.3	51.8	60.4	101.2	212.0	264.2
Feb	6.2	11.6	41.3	94.1	104.7	101.7	58.2	105.7	256.3	92.3
Mar	4.0	13.0	21.9	54.1	47.1	72.6	31.6	50.4	153.0	20.0
Apr	1.8	3.5	4.3	9.3	13.2	22.1	15.9	14.4	37.6	1.9
May	3.0	3.3	3.1	5.4	6.9	6.6	9.4	8.4	24.3	1.1
Jun	3.2	10.2	3.3	7.2	9.2	14.7	16.1	19.5	36.1	0.7
Jul	9.3	19.3	4.8	6.9	9.4	43.8	37.8	33.9	42.4	2.1
Aug	60.4	20.9	7.0	20.5	21.6	65.1	79.1	74.2	42.3	4.4
Sep	64.5	27.6	7.2	31.3	26.9	96.4	98.2	55.2	76.7	3.2
Oct	15.2	23.0	7.5	19.5	30.6	92.3	74.8	31.5	72.0	2.9
Nov	6.8	14.1	5.7	17.9	24.1	73.4	88.4	42.4	137.9	4.7
Dec	3.7	16.2	10.3	23.6	26.1	41.1	59.4	77.5	188.3	5.4
Annual	185.2	168.1	161.6	320.6	401.1	681.7	629.4	614.4	1,278.8	402.6
**Month**	**Incidence Rate of Pneumonia (per 100,000 Population)**									
Jan	39.0	54.8	48.4	54.2	36.9	45.7	64.4	68.2	58.6	69.8
Feb	35.6	51.7	52.2	56.4	43.0	47.0	64.5	68.5	54.0	51.1
Mar	29.6	52.7	47.1	43.0	43.7	50.4	58.1	69.2	47.8	39.7
Apr	24.7	35.6	37.4	36.5	32.5	42.1	43.9	49.1	42.2	26.2
May	25.2	29.7	31.4	28.9	29.4	36.0	44.4	46.9	40.7	22.2
Jun	23.6	31.7	22.3	26.2	31.1	38.3	47.6	51.0	40.1	18.3
Jul	27.2	34.2	24.6	25.1	30.2	44.5	39.7	50.0	39.2	20.3
Aug	49.3	44.5	28.8	31.4	50.3	72.6	55.9	58.1	39.3	24.1
Sep	59.5	57.1	44.4	51.4	55.0	82.6	66.4	70.4	53.3	25.2
Oct	50.6	41.1	39.5	40.1	55.2	56.1	65.6	60.9	57.9	28.6
Nov	42.9	29.4	31.7	29.1	46.3	47.8	57.1	53.7	54.7	40.1
Dec	34.3	23.2	23.3	30.4	43.6	41.9	49.0	51.9	59.0	38.9
Annual	441.4	485.7	431.2	452.7	496.9	605.0	656.7	697.8	586.9	404.6
